# Technostress, Burnout, and Job Satisfaction: An Empirical Study of STEM Teachers’ Well-Being and Performance

**DOI:** 10.3390/bs15070992

**Published:** 2025-07-21

**Authors:** Liya Tu, Zebo Rao, Haozhe Jiang, Ling Dai

**Affiliations:** 1 College of Education, Zhejiang University, Hangzhou 310058, China; liyatu@zju.edu.cn; 2 Faculty of Education, East China Normal University, Shanghai 200061, China; 52264101016@stu.ecnu.edu.cn; 3 National Institute of Education, Nanyang Technological University, Singapore 639798, Singapore

**Keywords:** technostress, STEM teachers, burnout, job satisfaction, commitment, work performance

## Abstract

This study investigates the creators, effects, and inhibitors of technostress among STEM teachers, addressing a critical yet underexplored issue in the digitalization of education. Grounded in the technostress model and the job demands–resources (JD-R) model, the study examines the relationships among technostress creators, burnout, organizational effects (job satisfaction, organizational commitment, and work performance), and technostress inhibitors. A cross-sectional survey was conducted with 378 STEM teachers from Zhejiang Province, China. Structural equation modeling (SEM) was employed to test the hypothesized paths. The results revealed that technostress creators significantly increased teacher burnout and negatively affected organizational commitment and work performance. Burnout mediated the impact of technostress creators on job satisfaction and organizational commitment. Technostress inhibitors were found to alleviate burnout, mitigate technostress creators, and enhance STEM teachers’ commitment. These findings validate the applicability of the technostress model in the context of K–12 STEM education in China and highlight the importance of organizational mechanisms for supporting teacher well-being and performance. The study contributes to both theory and practice by proposing an integrative model of technostress and offering actionable recommendations for school leadership to effectively manage technostress.

## 1. Introduction

### 1.1. Technostress: The Dark Side of Technology

Information and Communication Technology (ICT) has become deeply embedded in educational systems globally, significantly transforming the teaching and learning processes (Jiang et al., 2025). The proliferation of digital tools and platforms has enhanced communication, facilitated access to vast information resources, and fostered collaborative learning environments (Al-Fudail & Mellar, 2008; Jena, 2015). Previous research already reported that teachers are the key to ICT adoption in education (Williams et al., 2000; Shapka & Ferrari, 2003; Joo et al., 2016), for example, teachers’ ICT experiences and attitudes toward ICT use. When teachers feel confident with ICT, they are more likely to positively impact students’ learning (Almerich et al., 2016; Cacciamani et al., 2022). Hence, proficiency in ICT is now considered one of the key competencies required for teachers to succeed, as highlighted by frameworks such as the Technological Pedagogical Content Knowledge (TPACK) model (Mishra & Koehler, 2006).

However, despite the numerous advantages, the widespread integration of ICT into teaching is also associated with potential challenges, sometimes referred to as “crossing to the dark side” (Tarafdar et al., 2011). Issues such as increased workload, the need for constant upskilling, and the pressure to stay current with rapidly evolving technologies (Salanova et al., 2013; Henderson & Corry, 2021; Fernández-Batanero et al., 2021), collectively referred to as technostress, have emerged as a significant concern in educational research (Tarafdar et al., 2007; Ragu-Nathan et al., 2008). Technostress is a modern disease of adaptation caused by an inability to cope with new computer technologies in a healthy manner (Brod, 1984), and one of the consequences of an individual’s attempts and struggles to deal with constantly evolving ICT and the changing cognitive and social requirements related to their use (Ragu-Nathan et al., 2008; Chiappetta, 2017). While this study focuses on the negative impacts of technostress, it is important to acknowledge that technostress should be regarded as a holistic dynamic process involving both positive and negative aspects (Califf et al., 2020). Technostress can also serve as a source of motivation and positive challenge, inducing beneficial psychological responses and positive workplace outcomes (Nascimento et al., 2025). To maintain conceptual clarity, this study adopts a narrow definition of technostress, referring exclusively to its adverse effects.

### 1.2. STEM Teachers’ Well-Being and Performance

The specific demands of STEM (science, technology, engineering, and mathematics) teaching make STEM teachers particularly vulnerable to teacher stress (Jiang et al., 2021). Compared to traditional subject teachers, STEM teachers face pressures in all aspects of their work that cannot be ignored, because they need to have a wider range of competencies, including proficiency in disciplinary literacy and interdisciplinary knowledge. However, they face the fundamental dilemma of insufficient resources and a lack of support for innovation (Jiang et al., 2021). As a result, STEM teachers are subject to multiple pressures related to work, time, energy, and work support, as well as limitations from role load and job expectations (Eisenhart & Weis, 2022). Despite the increasing popularity of STEM teaching, empirical studies focusing on the technostress experienced by STEM teachers are still limited, and most studies on this topic focus on higher education contexts (Jena, 2015; Henderson & Corry, 2021; L. Li & Wang, 2021; Boufarouj, 2024).

Moreover, while previous studies have explored the negative effects of technostress, there is still some ambiguity regarding the relationship between individual-level and organizational-level effects (Tarafdar et al., 2011). Specifically, most of the existing literature has centered on organizational-level effects, including job satisfaction, organizational commitment, turnover, productivity, and so on (Jena, 2015; Joo et al., 2016; L. Li & Wang, 2021; Boufarouj, 2024; Aktan & Toraman, 2022), while overlooking the individual physiological and psychological effects on teachers, such as their burnout. Additionally, much of the current research has ignored the interaction between individual and organizational effects, highlighting the need for further investigation into how technostress at the individual level contributes to organizational-level effects.

Burnout has long been a critical area of study in educational psychology, defined as a state of emotional exhaustion, depersonalization, and reduced personal accomplishment resulting from prolonged work stress (Maslach & Jackson, 1982). The relationship between burnout and technostress, however, has not been extensively studied. While there is some research linking high technostress to burnout (Califf & Brooks, 2020; Panisoara et al., 2020), few studies have directly examined this relationship within the context of STEM teaching.

Furthermore, while technostress inhibitors (TIs), described as organizational mechanisms that have the potential to reduce the effects of technostress, have been suggested as potential solutions to mitigate the effects of technostress (Jena, 2015; Tarafdar et al., 2011; L. Li & Wang, 2021), their alleviative role is still not fully understood, particularly in how they interact with specific individual and organizational effects.

Motivated by the aforementioned gaps, this study investigates the creators, effects, and inhibitors of technostress within the context of STEM teaching. Grounded in the technostress model and the job demands–resources (JD-R) model, this study focuses on the role of burnout as a representative factor of individual effects of Technostress Creators (TCs). Furthermore, it examines job satisfaction (JS), commitment (CM), and work performance (WP) as representative factors reflecting the organizational-level impacts of technostress. Additionally, this study explores the mediating role of burnout between TC and organizational effects. A cross-sectional methodological approach is adopted, recruiting 378 STEM teachers from Zhejiang Province, China. This study aims to provide actionable insights into how TC influences STEM teachers’ burnout and JS, CM, WP, as well as TI’s contributions to improving STEM teachers’ JS, CM, WP and reducing burnout.

## 2. Theoretical Framework and Literature Review

### 2.1. Dimensions of Technostress Creators and Inhibitors

In this study, we examine four key dimensions of TC faced by STEM teachers based on (Ragu-Nathan et al., 2008; Tarafdar et al., 2011). The first dimension is Techno-overload (TOL), which occurs when increased technological demands escalate their workload, compelling them to work faster and take on more tasks than they can manage. The concept of techno-invasion (TIV), which describes the blurring of boundaries between work and personal life due to ICT, has been merged with TOL (L. Li & Wang, 2021). The second dimension is Techno-complexity (TCP), which refers to the difficulties STEM teachers encounter when using complex technological tools that are hard to master or integrate into their teaching practices. The third dimension is Techno-insecurity (TIS), which arises when STEM teachers feel insecure about their technological skills compared to their colleagues or fear being replaced by more tech-savvy individuals. The fourth dimension is Techno-uncertainty (TUC), which is related to the rapidly evolving nature of ICT in education. STEM teachers may struggle to keep up with frequent updates to software, hardware, and networks, creating uncertainty about their ability to stay current in an ever-changing technological landscape.

Several empirical studies have demonstrated that technostress inhibitors (TI) have a negative effect on technostress, with the majority focusing on the literacy facilitation dimension of TI. Ragu-Nathan et al. identified three distinct types of TI, each with its own definition (Ragu-Nathan et al., 2008). Specifically, literacy facilitation (LFL) refers to the opportunities provided for STEM teachers to share their ICT-related skills and knowledge within the organization. Technical support provision (TSP) involves the technical assistance offered by specialized personnel or departments to address STEM teachers’ ICT-related issues, thereby reducing the effects of technostress. Lastly, involvement facilitation (IVF) denotes the engagement of STEM teachers in the decision-making and implementation processes related to the integration of ICT in teaching and learning.

Prior research has investigated the effects of individual TI on specific TC (L. Li & Wang, 2021). Building upon this foundation, this study treats TC and TI as distinct yet integrated variables, exploring the individual effect (burnout) and specific organizational outcomes of technostress among K-12 STEM teachers. 

### 2.2. Transaction-Based (T-B) Approach and Technostress Model

The Transaction-Based (T-B) approach has long been a foundational framework for understanding stress in organizational research (Cooper et al., 2001). Unlike static or event-driven views of stress, this model emphasizes stress as a dynamic interaction between external stimuli and individuals’ internal evaluations and responses. It comprises four core elements: Stressors, Situational Factors, Strain, and Organizational Outcomes.

In the T-B model, if individuals perceive a situation as stressful and feel they lack the necessary resources, this appraisal leads to stress, which can result in various negative outcomes, such as burnout, reduced performance, and JS. Generally, Stressors intensify Strain, while Situational Factors help mitigate it. These situational mechanisms not only influence individual Strain but also potentially shape other Organizational Outcomes.

Grounded in the T-B approach, a technostress model that specifically addresses the stress arising from the use of ICT was developed (Ragu-Nathan et al., 2008). Each core component of the T-B approach is reflected in the technostress model. The main adaptation involved the redefinition of Stressors and Situational Factors by TC and TI, and subdividing their dimensions. TC corresponds to Stressors—external demands introduced by ICT use, TI aligns with Situational Factors, conceptualized as organizational resources that mitigate the impact of stressors and promote adaptive functioning.

A growing body of empirical research has applied and extended the technostress model, with its effectiveness being tested in different organizational work environments and multicultural contexts. Jena confirmed a strong relationship between TC, TI, and technostress effects in Indian academicians (Jena, 2015). Joo et al. found that school support had significant effects on technostress, and technostress significantly influenced Korean teachers’ intentions to use technology (Joo et al., 2016). A study confirmed the three-factor structure of the Italian technostress creators scale and highlighted that behavioral stress was positively correlated with workload, technostressors, and work–family conflict (Molino et al., 2020). The relationships among specific technostress inhibitors and specific creators have been investigated by using data collected from a Chinese university (L. Li & Wang, 2021).

However, despite these advancements, a notable gap exists in research on the STEM teacher population, with limited attention devoted to how technostress impacts this group. Furthermore, the relationship between individual effects of TC and organizational effects remains underexplored and warrants deeper investigation.

### 2.3. Job Demands–Resources (JD-R) Model

Burnout is a critical area of study in educational psychology. However, studies on the relationship between burnout and technostress remain insufficient. Several dimensions of TC (TOL, TIS, TIV) have been found to increase feelings of burnout among teachers (Califf & Brooks, 2020). The JD-R model provides an expanded perspective by viewing burnout as the result of the combined effect of both job demands and job resources (Demerouti et al., 2001). Job demands refer to the physical, psychological, social, or organizational aspects of a job that require sustained effort and are therefore associated with certain physiological or psychological costs. Job resources, in contrast, are the physical, psychological, social, or organizational aspects of the job that help achieve work goals, reduce job demands and their associated costs, or stimulate personal growth and development. The JD-R model posits two main processes: excessive job demands can lead to energy depletion, burnout, and health problems, while sufficient job resources foster engagement, increase motivation, and enhance performance.

According to the JD-R model, TC corresponds to job demands, and TI aligns with job resources. Excessive job demands can lead to burnout and organizational effects, while TI can help alleviate TC, burnout, and organizational effects.

### 2.4. Theoretical Framework for This Study

In this study, we primarily examine four effects of technostress: burnout (as individual effects) and JS, CM, and WP (as organizational effects), as well as the role of TI.

JS is defined as a pleasant or positive emotional state resulting from the evaluation of STEM teachers’ work or work experience (Ragu-Nathan et al., 2008; Aktan & Toraman, 2022; Ayyagari, 2012). From this perspective, it reflects not only attitudes toward the job but also the affective evaluation of work experiences (Long & Thean, 2011). Therefore, JS is one of the most frequently examined factors in the organizational behavior literature (Jena, 2015; Ragu-Nathan et al., 2008).

CM refers to STEM teachers’ attitude toward their schools, representing a psychological bond that influences the extent to which a teacher identifies with the school’s goals and values, exerts effort to achieve these goals, and desires to remain with the school (Allen & Meyer, 1990; Meyer et al., 1993). For STEM teachers, high levels of technostress reduce CM, as they may feel unsupported and unable to manage the increasing technological demands.

WP refers to how effectively a STEM teacher fulfills the requirements outlined in their job description (Borman & Motowidlo, 1997). Technostress negatively impacts WP by impairing STEM teachers’ ability to efficiently manage both teaching responsibilities and technology-related tasks. Moreover, ICT is increasingly becoming an integral part of education, thereby imposing higher demands on STEM teachers.

**Figure 1:**
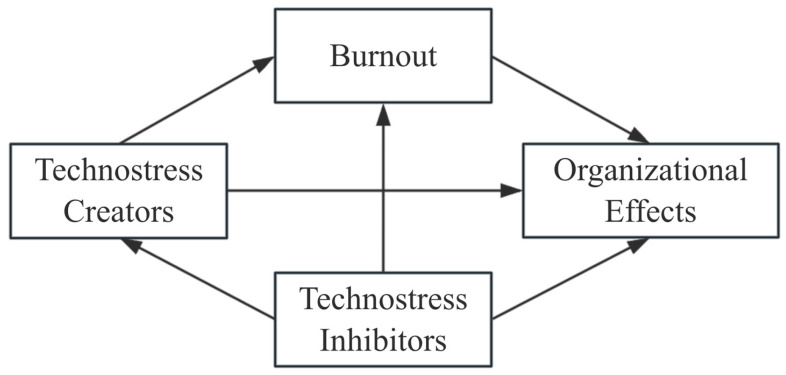
Theoretical framework for this study.

We present the theoretical framework for this study in Figure 1.

## 3. Hypotheses

### 3.1. Technostress and Organizational Effects

Technostress can lead to a wide range of adverse outcomes, including physical, psychological, social, and organizational effects (Atanasoff & Venable, 2017). Among these organizational effects, JS, CM, and WP have received considerable attention in the literature.

Studies have demonstrated that technostress negatively impacts JS. Specifically, sub-dimensions like TOL and TIS have been identified as variables that negatively affected JS (McDaniel et al., 2021; Aktan & Toraman, 2022). Moreover, TCP alone predicts both JS and teacher efficacy, indicating that TCP precedes other sub-dimensions. The key factor is how to understand the complexity of mobile technology (Lee & Lim, 2020). We hypothesize the following:

**Hypothesis 1.1 (H1.1).**  *TC will be negatively associated with STEM teachers’ JS.*

Many studies have identified the impact of technostress on CM, finding that TC leads to decreased organizational CM and continuance CM (Ragu-Nathan et al., 2008), which has been confirmed in the Indian collaborative learning environment (Jena, 2015) and among university teachers in Morocco (Boufarouj, 2024). A study from Malaysia revealed that TC jointly explained 13.1 percent of the variance in organizational CM among academic librarians (Ahmad et al., 2012). We hypothesize the following:

**Hypothesis 1.2 (H1.2).**  *TC will be negatively associated with STEM teachers’ CM.*

Productivity has been one of the adverse ICT use-related outcomes (Tarafdar et al., 2011). People suffering from a higher degree of TC have a greater likelihood of less technology-enabled performance (Jena, 2015). TCP and TIS have a significant negative influence on WP (L. Li & Wang, 2021); nevertheless, TOL is positively associated with WP, suggesting it may be an “enhancer” to one’s performance (Hung et al., 2015). We hypothesize the following:

**Hypothesis 1.3 (H1.3).**  *TC will be negatively associated with STEM teachers’ WP.*

Additionally, JS, CM, and WP also exhibit interdependent relationships. Some studies have suggested that CM positively influences employees’ JS (Canrinus et al., 2012; Zhao & Gao, 2014), while others argued that JS enhances employees’ CM (Heon & Lee, 2018). Meanwhile, both factors exerted a positive association with WP (Fu & Deshpande, 2014; Sesen & Basim, 2012).

### 3.2. Technostress and Burnout

Some empirical studies have further validated the applicability and validity of the JD-R model in cross-cultural contexts, and joint thematic studies have validated the applicability of this model among Chinese preschool teachers (Hong et al., 2021) and extended its application to investigate STEM teachers’ JS and work engagement (Z. Wang et al., 2024). As a form of stress, the impact mechanism of technostress can also be analyzed using the JD-R model. Therefore, based on the JD-R model, job demands in this study refer to the TC of STEM teachers, job resources refer to the TI of STEM teachers, and burnout is the result of the combined effect of TC and TI. We hypothesize the following:

**Hypothesis 2 (H2).**  *STEM teachers’ TC will be positively associated with burnout.*

### 3.3. Burnout and Organizational Effects

A large body of research conducted worldwide has revealed that burnout is a work-related phenomenon (Shoman et al., 2021; Agyapong et al., 2022; Mijakoski et al., 2022; Edú-Valsania et al., 2022), influencing both the quality of teacher performance and overall teacher well-being. Several studies support the relationship between burnout and key workplace outcomes such as CM and WP, indicating that burnout affects various aspects of the educational environment. Using the JD-R model, a study found that JS as an extrinsic motivation was negatively correlated with burnout (Panisoara et al., 2020). The job demands–control (JD-C) model posits that job strain results from the interaction between job demands and job control, and high demands coupled with low control are considered the most detrimental to employee well-being. Using the JD-C model, Martí-González et al. revealed that JS was negatively associated with emotional exhaustion and personal accomplishment—sub-dimensions of burnout (Martí-González et al., 2023). Burnout in teachers is associated with absenteeism, turnover intention, low JS, negative attitudes, and lack of interest in students and their education (D’Amico et al., 2020). Moreover, burnout has been identified as one of the determinants of teachers’ productivity or WP, as its symptoms negatively impact teacher motivation, teaching quality (Puertas Molero et al., 2019), and work efficiency (Lizano & Mor Barak, 2015). We hypothesize the following:

**Hypothesis 3.1 (H3.1).**  *STEM teachers’ burnout will be negatively associated with JS.*

**Hypothesis 3.2 (H3.2).**  *STEM teachers’ burnout will be negatively associated with CM.*

**Hypothesis 3.3 (H3.3).**  *STEM teachers’ burnout will be negatively associated with WP.*

### 3.4. The Mediation Role of Burnout

These associations highlight that burnout is not merely an individual health concern but a systemic organizational issue with implications for performance and retention in educational institutions. Burnout not only functions as a negative outcome of stressful work environments but also as a mediating mechanism linking stress to psychological dysfunction. For instance, a study examines the relationship between job stress, job burnout, JS, and organizational CM among university teachers in China, which found that job stress can positively predict organizational CM, and the independent mediating effect of job burnout is significantly greater than JS (P. Wang et al., 2020). Notably, these relationships have seldom been detected in the context of technostress. We hypothesize the following:

**Hypothesis 4.1 (H4.1).**  *STEM teachers’ burnout will mediate the impact of TC on JS.*

**Hypothesis 4.2 (H4.2).**  *STEM teachers’ burnout will mediate the impact of TC on CM.*

**Hypothesis 4.3 (H4.3).**  *STEM teachers’ burnout will mediate the impact of TC on WP.*

### 3.5. TI and Organizational Effects

In the educational environment, TI enhances STEM teachers’ experience with ICTs, increases their sense of competence and control, and is thus indicated to strengthen organizational outcomes. For instance, TI can offset the productivity-reducing effects of technostress by speeding up learning and decreasing users’ mistakes in the context of ICT use (Tarafdar et al., 2011). Jena found a significant relationship between TI and JS, organizational CM, negative affectivity, and technology-enabled performance (Jena, 2015). Specifically, both LFL and IVF were positively associated with university teachers’ WP (L. Li & Wang, 2021). We hypothesize the following:

**Hypothesis 5.1 (H5.1).**  *TI will be positively associated with STEM teachers’ JS.*

**Hypothesis 5.2 (H5.2).**  *TI will be positively associated with STEM teachers’ CM.*

**Hypothesis 5.3 (H5.3).**  *TI will be positively associated with STEM teachers’ WP.*

### 3.6. TI and Burnout

According to the JD-R model, teachers’ burnout is associated with school cultures and environments (Fernet et al., 2012). As a form of job resource, TI is expected to alleviate teacher burnout. For instance, it has been confirmed that LFL can mitigate the negative impact of burnout (Califf & Brooks, 2020). An investigation among teachers from Brazil found that LFL has an indirect effect on the perception of burnout (Malaquias & de Souza Junior, 2023). However, the alleviative role of TI in the context of STEM education remains underexplored. We hypothesize the following:

**Hypothesis 6 (H6).**  *TI will be negatively associated with STEM teachers’ burnout.*

### 3.7. TI and TC

With regard to technostress, research findings suggest that support from teacher community or from schools—such as opportunities for communication and knowledge sharing related to the use of new technologies and open educational resources—is essential for reducing teachers’ technostress (Hasan Özgür, 2020; Zhao & Gao, 2014). Conversely, insufficient support from schools may lead to an increasing technostress of teachers (Joo et al., 2016). Additionally, some studies confirmed specific dimension of TI will impact TC. For example, LFL (e.g., training, instruction materials, and knowledge sharing) has a negative effect on the perceptions of TOL, TCP, and TIS (Malaquias & de Souza Junior, 2023). It can also mitigate the negative impact of most dimensions of TC except TUC (Califf & Brooks, 2020). Furthermore, TSP had particularly curbing effects on TOL, TCP, and TIS (L. Li & Wang, 2021). We hypothesize the following:

**Hypothesis 7 (H7).**  *TI will be negatively associated with TC.*

**Figure 2:**
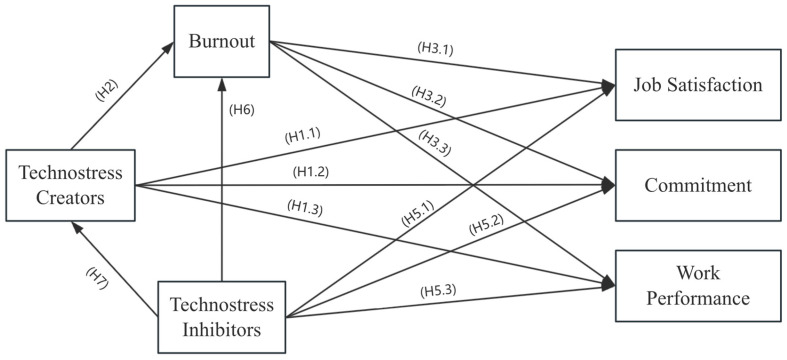
Research model.

Drawing on the previous literature on technostress creators, effects, and inhibitors, we present our conceptual research model (Figure 2) to elaborate on the hypotheses associated with the research model.

## 4. Materials and Methods

This study aims to investigate the creators, effects, and inhibitors of technostress among STEM teachers. Given the complex nature of technostress and its various effects, a quantitative cross-sectional survey design is considered the most appropriate method for this research. This approach enables the collection of data from a large sample of teachers in a structured and standardized manner, thereby facilitating the examination of relationships between elements, providing a reliable way to assess the theoretical framework and hypotheses presented above.

### 4.1. Participants

The participants of this study are STEM teachers from Zhejiang Province, located in eastern China. Zhejiang participated in the PISA 2018 testing, representing China (B-S-J-Z), and achieved top global rankings, particularly in mathematics and science (OECD, 2019). Zhejiang’s exceptional performance in PISA assessments in the fields of mathematics and science is indicative of the region’s high academic standards and rigorous STEM education system. Moreover, Zhejiang is a pioneering and exemplary province in China’s education reform. China follows a national education standards system administered by the Ministry of Education. While local innovations may occur, especially in a relatively advanced province like Zhejiang, these operate within a unified national framework. Zhejiang has introduced science curricula starting from the first grade of primary school. In middle school, science curricula integrate various disciplines, offering comprehensive science courses that combine physics, chemistry, and biology, rather than teaching them as separate subjects (Department of Education of Zhejiang Province, 2023). At the high school level, the curriculum includes a technology subject, combining information technology and general technology, as a part of the National College Entrance Examination (The People’s Government of Zhejiang Province, 2014). Notably, while engineering is not taught as a standalone subject, engineering concepts are integrated into mathematics, science, and technology instruction.

In total, 459 teachers participated in the survey. After an initial check, we removed 81 invalid responses, and the effective rate is 82.3%. Among the 378 effective responses, 103 (27.2%) participants were male STEM teachers, and 273 (72.8%) participants were female. The gender split is a representative sample of teachers in Zhejiang Province (69.5% teachers in basic education are female) (Department of Education of Zhejiang Province, 2025). Participants’ age ranged from 25 to 50 years, with the majority being middle-aged teachers (between 30 and 50 years old, 257 participants, 61%). Teaching experience of participants varied, with 72.2% having 5–30 years of teaching experience, and 21.4% being novice teachers (less than 5 years). A total of 230 participants (60.8%) were from primary schools, 59 (15.6%) from middle schools, 73 (14.3%) from high schools, and 16 (4.2%) from other K-12 integrated schools.

The sample represented a diverse array of subjects, including mathematics, science, and technology: a total of 67 (17.7%) participants were mathematics teachers, 207 (54.8%) participants were science teachers, and 62 (16.4%) participants were technology teachers, while only 42 (11.1%) teachers specialized in teaching integrated STEM courses. Additionally, 356 (94.2%) participants had experience implementing integrated STEM teaching in schools, and the schools of 167 (44.2%) participants were equipped with ICT professional technical personnel or a dedicated department.

### 4.2. Instrument Development

The study involved six instruments, which were used to measure STEM teachers’ TC, TI, burnout, and organizational effects (JS, CM, WP), respectively. The scale for measuring TC and TI was adapted from the technostress scale (L. Li & Wang, 2021; Ragu-Nathan et al., 2008). The scale for measuring organizational effects has three sub-scales: the JS scale was adapted from (Ho & Au, 2006), for its better alignment with China’s linguistic and cultural context, the CM scale was adapted from (Allen & Meyer, 1990), and the WP scale was adapted from (Torkzadeh & Doll, 1999). The scale for measuring burnout was adapted from the Copenhagen Burnout Inventory (Kristensen et al., 2005), which distinguishes between personal, work-related, and client-related burnout, offering more domain-specific measurement. The final version of the instrument had 42 items, which were designed using a five-point Likert scale, with “1” representing “strongly disagree” and “5” representing “strongly agree” (see Appendix A). To ensure data quality, an instructional manipulation check was embedded in the questionnaire. The item explicitly instructed participants to select “agree” as their response. Failure to follow this instruction was considered an indication of inattentive or careless responding.

The questionnaire was developed based on established scales used in previous research on technostress, adapted to the Chinese educational context. To ensure clarity and eliminate ambiguity, the questionnaire was initially translated into Chinese by two educational experts who were proficient in both English and Chinese, and then reviewed and adjusted by a group of two education specialists and five STEM teachers.

The questionnaire underwent a pilot test with a smaller group of 200 STEM teachers to evaluate its clarity, reliability, and validity. Based on feedback data from SPSS version 27 and AMOS version 26, some items were deleted to improve the instrument’s construct validity and internal consistency. The 200 STEM teachers who participated in the pilot test were included in the final sample, as no major changes were made to the survey instrument following the pilot test. 

### 4.3. Data Collection and Data Analysis

The data collection was conducted through an online survey platform www.wjx.cn (accessed on 17 May 2025), which allowed for anonymous responses from the participants. This study had been approved by the school ethics review committee, and ethical considerations were taken into account throughout the process by adhering to the Declaration of Helsinki. Informed consent was obtained from all participants before they completed the questionnaire, ensuring that they understood the purpose of the study, their voluntary participation, and their right to withdraw at any time without penalty. The survey was designed to be completely anonymous, and no personal identifiers were collected to maintain participant privacy. Moreover, participants were assured that their responses would not affect their professional evaluations or work situations.

The data were analyzed using structural equation modeling (SEM), which is suitable for examining complex relationships between observed and latent variables (Hoyle, 1995). The analysis was conducted using SPSS 27 and AMOS 26, both of which are commonly used software packages for structural equation modeling (Byrne, 2016; Pallant, 2020). The first step involved conducting a measurement model to test the validity and reliability of the constructs. After confirming the measurement model, the next step involved testing the structural model to examine the hypothesized relationships between the variables.

## 5. Results

In this section, we first provide a concise and precise description of the questionnaire results. Subsequently, the results of the structural model are presented to scrutinize our hypotheses.

### 5.1. Reliability and Validity of the Questionnaire

The questionnaire was tested for reliability using Cronbach’s alpha, The reliability tests showed strong results, with Cronbach’s alpha ranging from 0.758 to 0.904 (TC:0.883; TI:0.904; burnout:0.884; JS:0.758; CM:0.828; WP:0.847), all of which are above the accepted value of 0.70, indicating that the questionnaire had sufficient reliability (Hair et al., 2013).

Goodness-of-fit indices such as CFI, TLI, and RMSEA were used to assess the overall fit of the model. According to (Hair et al., 2013), models with CFI ≥ 0.90, TLI ≥ 0.90, and RMSEA ≤ 0.08 are considered to have a good fit. The structural model fitted the data well with CFI = 0.946, TLI = 0.942, and RMSEA = 0.051.

Item reliability is assured when all items’ factor loadings are above 0.70 (Hair et al., 2013). As shown in Table 1, the factor loadings of most items met this requirement. Convergent validity was achieved by satisfying two criteria: (1) the composite reliability (CR) of all constructs should exceed 0.70; and (2) the average variance extracted (AVE) of each construct should be above 0.50 (Hair et al., 2013). As indicated in Table 1, the constructs’ CR coefficients were between 0.881 and 0.981, and the AVE values for each construct exceeded 0.50, ranging from 0.712 to 0.855. Therefore, our instruments possessed good convergent validity.

In addition, discriminant validity was examined using two criteria: the square root of AVE for each construct should be greater than the correlation coefficients between that construct and other constructs, and each item should have higher loadings on its associated construct than on other constructs (Hair et al., 2013). Table 2 indicates that the criteria of discriminant validity were met in this study.

In summary, by conducting the CFA and validating the measurement model, the validity and reliability of our questionnaire were successfully established.

### 5.2. Results of the Structural Model

**Figure 3:**
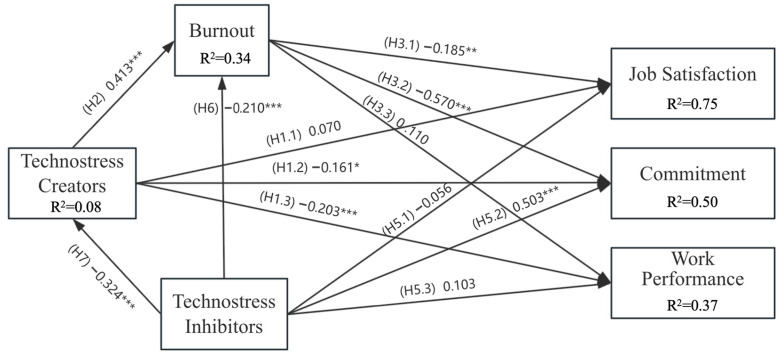
Results of hypotheses testing (H1–3, H5–7). *: *p* < 0.05, **: *p* < 0.01, ***: *p* < 0.001.

Figure 3 and Table 3 present the results of hypotheses testing (H1–3, H5–7). TC had a significant negative effect on STEM teachers’ CM (β = −0.161, *p* = 0.015) and WP (β = −0.203, *p* < 0.001) and a significant positive effect on burnout (β = 0.413, *p* < 0.001). STEM teachers’ burnout had a significant negative effect on JS (β = −0.185, *p* = 0.002) and CM (β = −0.570, *p* < 0.001). Furthermore, TI had a significant negative effect on TC (β = −0.324, *p* < 0.001), a positive effect on STEM teachers’ CM (β = 0.503, *p* < 0.001), and a negative effect on burnout (β = −0.210, *p* < 0.001). Therefore, H1.2, H1.3, H2, H3.1, H5.2, H6, and H7 were accepted.

Contrary to expectations, the impact of TC on STEM teachers’ JS was not significant (β = 0.070, *p* = 0.099). STEM teachers’ burnout did not demonstrate a significant effect on WP (β = 0.110, *p* = 0.191). Additionally, TI was not significantly associated with STEM teachers’ JS (β = −0.056, *p* = 0.283) and CM (β = 0.103, *p* = 0.075). As such, H1.1, H3.3, H5.1 and H5.3 should be rejected.

As shown in Figure 3, the model explains a substantial proportion of variance in the endogenous constructs. Specifically, the R^2^ values of TC, burnout, JS, CM, and WP were 0.08, 0.34, 0.75, 0.50, and 0.37, respectively, indicating moderate to substantial predictive relevance (Cohen, 2013).

Table 4 shows the results of hypotheses testing (H4), where mediating effects were involved. Burnout significantly mediated the impact of TC on JS and CM (β = −0.076, *p* = 0.012; β = −0.235, *p* < 0.001); H4.1 and H4.2 were supported. Notably, when burnout was introduced as a mediating variable, the direct relationship between TC and JS became nonsignificant, indicating that burnout fully mediated the relationship between TC and JS, signifying a complete mediation effect. However, the indirect impact of TC on WP via burnout was not significant (β = 0.045, *p* = 0.210). Therefore, H4.3 was rejected.

## 6. Discussion

### 6.1. Theoretical Contribution

This study makes several key theoretical contributions to the literature on technostress within the context of STEM education. As STEM teaching increasingly becomes a central pillar in 21st-century education reform, understanding the physical and psychological pressures faced by STEM teachers is essential for ensuring sustainable pedagogical innovation and teacher retention. STEM educators are uniquely positioned at the intersection of interdisciplinary knowledge demands, technological competence, and innovation expectations (Eisenhart & Weis, 2022). Our findings highlight the pressures experienced by STEM teachers, reinforcing the urgency to examine technostress not merely as a technological issue but as an occupational phenomenon with profound implications for educational quality and teacher well-being.

Descriptive statistics of our large-scale survey (N = 378) revealed that STEM teachers reported a moderate level of TC, with most item means clustering around 3.00. TI received moderately high ratings, with several items exceeding 3.5, suggesting schools’ widespread emphasis on ICT skills training, technical support, and incentives for teachers. Burnout responses were generally moderate to low, with personal burnout (PB) items scoring higher than others. JS, CM, and WP were rated relatively high, with most values above 3.5, especially WP. Overall, the data reflect a balanced profile of technostress functioning in STEM teaching contexts.

Analysis across school levels revealed that high school teachers reported the highest levels of TC (M = 3.32) and burnout (M = 3.03), despite also perceiving the strongest TI (M = 3.99). This may suggest a match between stressors and support effectiveness at this level. Primary school teachers experienced the lowest TC (M = 3.03) and burnout (M = 2.76), but reported the second highest access to TI (M = 3.50), possibly due to simpler ICT demands or more institutional support. Middle school teachers showed moderate levels across all three variables: TC (M = 3.11), TI (M = 3.38), and burnout (M = 2.82). These differences highlight the importance of tailoring intervention strategies by educational stage.

Furthermore, this study empirically revalidates the technostress model within the cultural and institutional context of K-12 STEM education in China (Ragu-Nathan et al., 2008). Our findings confirmed its relevance in Chinese primary and secondary schools, particularly within STEM disciplines. Consistent with theoretical expectations, TC significantly predicted higher levels of burnout among STEM teachers (H2), confirming that frequent exposure to technostress overload, complexity, insecurity, and uncertainty contributes to burnout. This finding aligns with prior research (Califf & Brooks, 2020), reinforcing the notion that in the context of STEM teaching—where digital tools are heavily embedded—teachers are vulnerable to stress accumulation, especially in environments lacking sufficient adaptive support.

As expected, TC significantly reduced organizational CM (H1.2) and perceived work performance (H1.3), consistent with the technostress model’s proposition that stressors weaken employees’ engagement with their institutions and impair performance. However, TC did not significantly reduce JS (H1.1). This may reflect a culturally specific resilience among Chinese STEM educators, who often demonstrate professional obligation and adaptive capacity despite high workloads.

Regarding the effects of burnout, the results demonstrated that burnout significantly undermined JS (H3.1) and CM (H3.2), which supported the model’s strain-outcome logic. However, the study did not find a statistically significant association between STEM teachers’ burnout and self-reported WP. It is important to acknowledge how WP was conceptualized and measured in this study. Rather than assessing objective behavioral performance, the items of WP reflected teachers’ subjective beliefs regarding the extent to which ICT use enhances their professional functioning. As such, this measure may be more indicative of their cognitive or attitudinal expectations rather than actual performance outcomes, potentially attenuating the observed effect of burnout. While ICT may bring burnout, its role in enhancing productivity is undoubtedly undeniable. One plausible explanation lies in social and cultural factors, particularly within the Chinese education system. In recent decades, China has aggressively promoted educational informatization as a national strategy, with strong top-down support from governmental policies and public discourse that emphasizes the transformative potential of ICT in teaching. The government can serve as a driving force to develop and diffuse ICT into education by taking a regulatory and coordinating role (Verdegem & Verhoest, 2009). In such a context, teachers may feel compelled to report confidence in technology-enhanced performance (Y. Li et al., 2019).

Moreover, mediation analyses confirmed that burnout mediated the effect of TC on JS and CM, offering insights into the psychological mechanisms through which technostress affects organizational outcomes. These findings underscore the central role of psychological effects in educational settings as a transmission channel linking environmental stressors and organizational outcomes.

TI demonstrated a significant negative relationship with burnout (H6) and TC (H7), as well as a positive effect on CM (H5.2), validating their role as protective organizational resources (Ragu-Nathan et al., 2008). However, the nonsignificant influence of TI on JS (H5.1) and WP (H5.3) suggested that while TI can buffer emotional strain and foster organizational belonging, it may not fully translate into improvements in evaluative or productivity-related outcomes unless coupled with broader systemic reforms or teacher empowerment mechanisms.

Collectively, this study bridges three major research gaps identified in prior literature. Firstly, by focusing on STEM teachers in the K-12 context, we provided empirical insights into an understudied population. Secondly, we advanced the studies of technostress by confirming the mediating role of burnout in the relationship between TC and organizational effects. Thirdly, we highlighted the role of TI—organizational-level buffers that mitigate the negative consequences of TC.

### 6.2. Practical Implications

Building on the validated structural model, this study offers targeted recommendations to help schools mitigate technostress and enhance organizational outcomes among STEM teachers.

Firstly, the demonstrated link between technostress and burnout, as well as diminished CM and WP, highlights the urgent need to incorporate digital resilience training into professional development programs. Training initiatives should equip teachers not only with technical skills but also with strategies to manage stress and adapt to rapid technological change. Schools should foster teachers’ collaborative professional learning communities (Ryymin et al., 2008; Vangrieken et al., 2015), which can serve as platforms for teachers to share experiences, strategies, and resources related to ICT integration, thereby reducing feelings of isolation and enhancing collective efficacy. Such communities have been shown to alleviate technostress by promoting peer support and shared problem-solving (Califf & Brooks, 2020; Khlaif et al., 2023; Malaquias & de Souza Junior, 2023). In Chinese schools, a practical approach is to embed collaborative professional learning communities into routine school operations—for example, by mandating a “Teaching and Researching Group” within each teaching team (L. Wang, 2020). More experienced or digitally skilled teachers can be formally assigned to mentor others, with incentives tied to this role. Furthermore, administrative leaders can incorporate ICT well-being into regular meetings, ensuring that technostress is addressed as a shared organizational concern rather than an individual issue (Zhang & Pang, 2016).

Secondly, the study confirms that burnout mediates the relationship between technostress and JS, CM. Therefore, education policymakers should consider implementing institutional supports such as targeted emotional regulation trainings (Farhi & Rubinsten, 2024), including mindfulness, stress management techniques, and resilience-building exercises should be implemented in schools. These interventions can equip teachers to better cope with the emotional demands of technology integration.

Thirdly, schools should invest in robust technical support systems. This includes ensuring timely assistance with ICT issues, developing clear documentation, and offering ongoing training sessions. Moreover, involving teachers in the selection, implementation, and evaluation of educational technologies can enhance their sense of ownership and reduce resistance to change. This participatory approach not only aligns technological initiatives with teachers’ needs and capacities but also fosters a more supportive organizational climate, thereby effectively mitigating technostress (Khlaif et al., 2023; L. Li & Wang, 2021; Tarafdar et al., 2011).

### 6.3. Limitations and Future Research

While this study provides valuable insights into the mechanisms and consequences of technostress among STEM teachers, it still has certain limitations. These limitations, some inherent to the research design and others related to theoretical scope, present opportunities for refinement in future investigations. Firstly, our study employed a cross-sectional survey method, which inherently restricts the ability to draw causal inferences as all data were collected at a single time point, and the findings should therefore be interpreted as preliminary associations rather than definitive directional effects. Teachers’ emotional states, such as burnout or JS, may fluctuate across academic semesters or in response to policy and technological changes. Relying on cross-sectional data presented specific challenges for mediation analysis (Maxwell & Cole, 2007; Mitchell & Maxwell, 2013; O’Laughlin et al., 2018). Longitudinal research designs would allow future studies to examine how technostress evolves over time and whether the effects of TI are sustained or vary with contextual change.

Secondly, this study did not examine the individual effects of each TC and TI. While the model treated these constructs as aggregated dimensions, prior literature has highlighted that specific types of TC may exert differential impacts on psychological and organizational effects. Similarly, individual TI may vary in their buffering capacity. Future research could disaggregate these dimensions to explore their unique pathways and interactions, offering a more nuanced understanding of how different forms of technostress and their mitigation strategies influence burnout, JS, and WP. Moreover, this study treated ICT as a generalized construct without distinguishing between types of technologies. However, different technologies may trigger different dimensions or intensities of technostress. Future research would benefit from employing mixed methods, such as qualitative interviews or classroom observations, to explore how specific technologies contribute uniquely to technostress and how teachers cope with them in context-sensitive ways (Palak & Walls, 2009).

Thirdly, the study focused on a single province (Zhejiang) within China. Given the regional variations in ICT infrastructure, administrative policies, and teacher development systems across provinces, generalizing these findings to the national level requires caution. Comparative studies across different provinces or between urban and rural school systems, and between primary, middle, and high school systems could yield a more comprehensive understanding of how local education cultures influence technostress dynamics.

Finally, while this study focused on burnout as the central individual outcome, expanding the outcome scope to include strain, cognitive fatigue, or innovation resistance in future work could be fruitful. Such variables could provide deeper insight into the psychosocial and pedagogical costs of digital transformation in schools.

## 7. Conclusions

This study provides empirical evidence on the detrimental impact of technostress on STEM teachers’ well-being and performance. Specifically, it demonstrates that technostress significantly increases burnout while simultaneously reducing commitment and work performance. Importantly, the study highlights the mitigating role of technostress inhibitors, which act as protective resources that alleviate the negative psychological and organizational effects of technostress.

By validating and extending the technostress framework within the Chinese K–12 educational system—a context underrepresented in the existing literature—this research advances both theoretical understanding and practical implications. It bridges individual-level and organizational-level effects, reinforcing the urgency of designing policies and interventions that support digital resilience among teachers. The findings underscore the need for targeted professional development, stronger technical support structures, and proactive strategies in education policy to ensure that the growing integration of digital tools enhances rather than impairs teacher well-being and performance.

In an era where digitalization in education is accelerating globally, this study offers timely, actionable insights for educators, administrators, and policymakers seeking to sustain teacher motivation, engagement, and retention in technology-rich teaching environments.

## Figures and Tables

**Table 1:** Reliability, average variance extracted (AVE), and factor loadings (*N* = 378).

Constructs/Items	AVE	CR	Factor Loadings	M	SD
Technostress Creators	0.722	0.971			
TOL1			0.832	3.17	1.108
TOL2			0.854	3.12	1.122
TOL3			0.891	3.28	1.124
TOL4			0.869	3.10	1.086
TCP1			0.913	3.06	1.041
TCP2			0.946	3.10	1.033
TCP3			0.812	2.88	1.064
TIS1			0.875	2.74	0.999
TIS2			0.728	3.73	0.991
TIS3			0.660	2.60	0.954
TUC1			0.894	2.99	0.907
TUC2			0.944	3.03	0.911
TUC3			0.775	2.84	0.904
Burnout	0.855	0.981			
PB1			0.951	3.37	0.896
PB2			0.958	3.39	0.936
PB3			0.911	3.28	0.967
WB1			0.952	2.85	1.057
WB2			0.916	2.61	1.017
WB3			0.951	2.81	1.077
CB1			0.765	2.13	0.939
CB2			0.950	2.15	0.914
CB3			0.952	2.22	0.976
Job Satisfaction	0.771	0.910			
JS1			0.883	3.74	0.788
JS2			0.847	3.72	0.791
JS3			0.904	3.54	0.849
Commitment	0.712	0.881			
CM1			0.880	3.60	0.894
CM2			0.820	3.51	0.931
CM3			0.831	3.61	0.901
Work Performance	0.782	0.935			
WP1			0.836	3.83	0.764
WP2			0.896	3.84	0.777
WP3			0.879	3.79	0.814
WP4			0.924	3.88	0.786
Technostress Inhibitors	0.720	0.962			
LFL1			0.914	3.55	0.882
LFL2			0.811	3.74	0.877
LFL3			0.841	3.44	0.976
TSP1			0.906	3.33	0.955
TSP2			0.889	3.24	0.975
TSP3			0.909	3.41	0.976
TSP4			0.898	3.41	0.940
IVF1			0.786	3.36	0.942
IVF2			0.862	3.54	0.868
IVF3			0.625	3.84	0.766

**Table 2:** Discriminant validity (*N* = 378).

Constructs	TC	Burnout	JS	CM	WP	TI
TC	**0.850**					
Burnout	0.413 ***	**0.925**				
JS	−0.070	−0.185 **	**0.878**			
CM	−0.161 *	−0.570 ***	0.669 ***	**0.844**		
WP	−0.203 ***	0.110	−0.446 ***	0. 029	**0.884**	
TI	−0.324 ***	−0.210 ***	−0. 056	0.503 ***	0.103	**0.849**

*: *p* < 0.05, **: *p* < 0.01, ***: *p* < 0.001. The bold numbers refer to the square roots of the AVE values.

**Table 3:** Results of hypotheses testing (H1–3, H5–7).

Hypothesis	Path	Coefficients	SE	C.R.	Result
H1.1	TC→JS	0.070	0.042	−1.648	Reject
H1.2	TC→CM	−0.161 *	0.066	2.427	Accept
H1.3	TC→WP	−0.203 ***	0.051	−4.003	Accept
H2	TC→burnout	0.413 ***	0.056	7.432	Accept
H3.1	burnout→JS	−0.185 **	0.059	−3.106	Accept
H3.2	burnout→CM	−0.570 ***	0.078	−7.287	Accept
H3.3	burnout→WP	0.110	0.084	1.309	Reject
H5.1	TI→JS	−0.056	0.052	−1.073	Reject
H5.2	TI→CM	0.503 ***	0.074	6.838	Accept
H5.3	TI→WP	0.103	0.058	1.782	Reject
H6	TI→burnout	−0.210 ***	0.055	−3.838	Accept
H7	TI→TC	−0.324 ***	0.072	−4.479	Accept

*: *p* < 0.05, **: *p* < 0.01, ***: *p* < 0.001.

**Table 4:** Results of hypotheses testing (H4).

Hypothesis	Path	Coefficients	95%CI	Result
H4.1	TC→burnout→JS	−0.076 *	[−0.164, −0.017]	Accept
H4.2	TC→burnout→CM	−0.235 ***	[−0.354, −0.151]	Accept
H4.3	TC→burnout→WP	0.044	[−0.028, 0.130]	Reject

*: *p* < 0.05, ***: *p* < 0.001.

## Data Availability

The data presented in this study are available on request from the corresponding author due to confidentiality and privacy reasons.

## Appendix A

**Table A1:** The items of the questionnaire.

Instrument	Items	Factor Loadings	Description
**Technostress Creators**			
Techno-overload	TOL1	0.832	Because of ICT, I had to schedule my work under a very tight schedule.
	TOL2	0.854	I was forced to change my work habits to adapt to teaching under new technology.
	TOL3	0.891	My workload has increased because of the increase in technical complexity.
	TOL4	0.869	I have to work faster because of ICT.
	TOL5 *	0.502	I have a higher workload because of the increased complexity of ICT.
Techno- complexity	TCP1	0.913	I often find that ICT is too complicated, and I can’t understand it well.
	TCP2	0.946	I often find that ICT is so complex that I can’t use it effectively.
	TCP3	0.812	The complexity of ICT makes me doubt its usefulness in education.
Techno- insecurity	TIS1	0.875	ICT disrupted my normal working mode.
	TIS2	0.728	I have to constantly upgrade my skills to avoid being replaced by new technology one day.
	TIS3	0.660	My job security is constantly under threat due to the constant introduction of new technologies in my school.
	TIS4 *	0.599	I am threatened by colleagues with stronger ICT skills.
Techno- uncertainty	TUC1	0.894	The technology used in our school is frequently upgraded.
	TUC2	0.944	The technology used in our school, the functions of which are constantly changing.
	TUC3	0.775	Our school often substitutes one technology for another.
**Technostress Inhibitors**			
Literacy facilitation	LFL1	0.914	Our school provides adequate professional training to ensure that we use technology effectively.
	LFL2	0.811	Our school focuses on teamwork to promote the use of technology.
	LFL3	0.841	Our school has developed effective documentation to help teachers use technology.
Technical support provision	TSP1	0.906	Our school’s technical support department does a great job of answering questions about the use of technology.
	TSP2	0.889	Our school’s technical support department is made up of knowledgeable personnel.
	TSP3	0.909	Our school’s technical support department is easy to communicate.
	TSP4	0.898	Our school’s technical support department will respond to our requests at any time.
Involvement facilitation	IVF1	0.786	Teachers were consulted before the technology was put into use.
	IVF2	0.862	Teachers are involved in the transformation and promotion of new technologies.
	IVF3	0.625	Our school allows teachers the freedom to choose the technology they use.
	IVF4 *	0.479	We are rewarded for using ICT in our daily work.
**Organizational Effects**			
Job satisfaction	JS1	0.883	I like to do what I do at work.
	JS2	0.847	I’m proud of my work.
	JS3	0.904	I’m having a good job.
Commitment	CM1	0.880	I would love to spend the rest of my career in this school.
	CM2	0.820	This school has a lot of personal meaning to me.
	CM3	0.831	Staying at the school where I currently work is something that I think is necessary and desired.
	CM4 *	0.447	I think the school thing is my thing.
	CM5 *	0.605	If I decide to leave the school where I currently work immediately, my life will be disrupted too much.
	CM6 *	0.442	Even if I want to leave the school where I currently work, it will be difficult in the short term.
Work performance	WP1	0.836	The use of technology has improved my productivity.
	WP2	0.896	The use of technology has improved the quality of my work.
	WP3	0.879	Technology has enabled me to do things that were previously difficult to do.
	WP4	0.924	Technology has enabled me to experiment with innovative ideas.
**Burnout**			
Personal burnout	PB1	0.951	I feel tired.
	PB2	0.958	I feel physically exhausted.
	PB3	0.911	I feel emotionally exhausted.
Work- related burnout	WB1	0.952	Work makes me feel emotionally exhausted.
	WB2	0.916	Work frustrates me.
	WB3	0.951	I felt burned out because of my work.
Client- related burnout	CB1	0.765	Communicating with students drains my energy.
	CB2	0.950	Communicating with colleagues drains my energy.
	CB3	0.952	When I work with my colleagues, I give more than I get.
	CB4 *	0.534	I’m tired of communicating with students.
	CB5 *	0.525	I find it difficult to communicate with my colleagues.

* Items were deleted after factor analyses, and factor loadings for other items are recalculated after removing items with asterisks.
